# Endoscopic ultrasound-guided dual-route recanalization of isolated hepatic ducts following complete hilar disconnection

**DOI:** 10.1055/a-2781-5937

**Published:** 2026-02-05

**Authors:** Jianbo Ni, Guanhao Su, Kai Xu, Xiaoyuan Gong, Baiwen Li

**Affiliations:** 1 Department of Gastroenterology and Digestive Endoscopy, Shanghai General Hospital, Shanghai Jiao Tong University School of Medicine, Shanghai, China

**Fig. 1:**
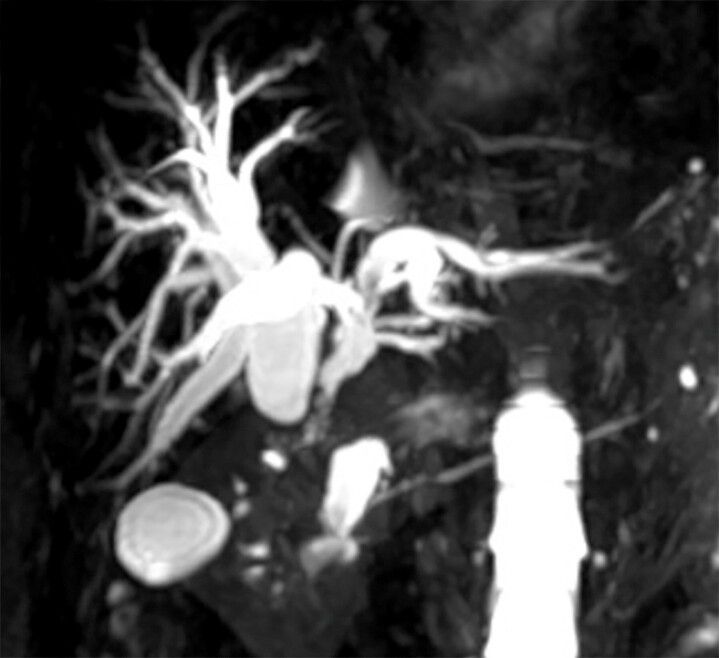
MRCP demonstrating complete hilar disconnection with abrupt truncation of the common bile duct and absent intrahepatic opacification. MRCP, magnetic resonance cholangiopancreatography.

Management of biliary-enteric anastomotic occlusion, especially in cases of complete hilar disconnection, is challenging. Endoscopic ultrasound (EUS)-guided biliary drainage is now a key alternative to endoscopic retrograde cholangiopancreatography (ERCP), providing reliable transluminal access for managing complex biliary obstruction 1 2 3 . This case reported a 72-year-old female patient presented with fever and progressive obstructive jaundice following aprior biliary surgery. Magnetic resonance cholangiopancreatography revealed the complete separation of the right and left hepatic ducts with abrupt truncation of the common bile duct and non-opacification of intrahepatic branches ( Fig. 1 ). Conventional ERCP failed as contrast injection only visualized a short blind-ending common bile duct.

**Fig. 2:**
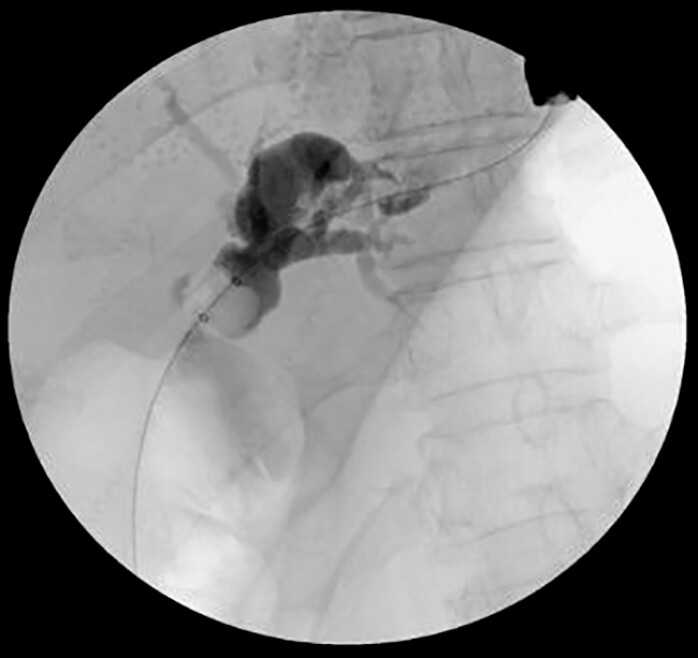
EUS-guided left hepaticogastrostomy for left intrahepatic biliary drainage. EUS, endoscopic ultrasound.

To decompress the left hepatic system, endoscopic ultrasound-guided hepaticogastrostomy (EUS-HGS) was performed. A 19-G needle was used to puncture a dilated left intrahepatic duct from the gastric wall, followed by guidewire advancement and dilation of the puncture tract. Balloon dilation of the anastomotic stricture was performed first, followed by antegrade balloon stone removal ( Fig. 2 ). Cholangioscopic examination confirmed the resolution of the stenosis, allowing bile to flow freely into the jejunum and leading to the resolution of cholangitis symptoms.

**Fig. 3:**
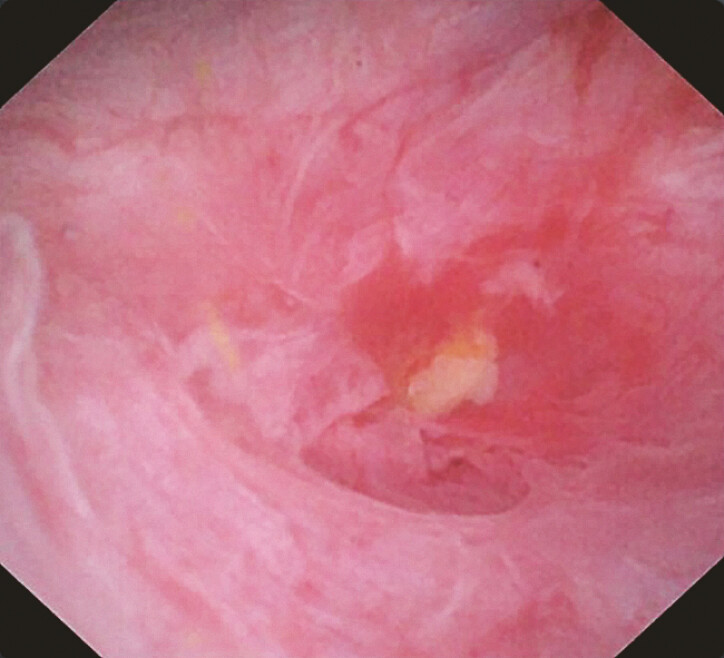
Direct cholangioscopic examination via the PTCD tract. PTCD, percutaneous transhepatic cholangiography and drainage.

EUS-guided biliary drainage of the right hepatic system could not be performed due to its excessive distance and anatomical inaccessibility. A percutaneous transhepatic cholangiography and drainage (PTCD) catheter was maintained for right hepatic drainage and further exploration. Direct cholangioscopy via the PTCD tract revealed complete closure of the right intrahepatic duct, which appeared as a blind end ( Fig. 3 ). An attempt to puncture the duct with a modified 22G EUS-FNA needle (with sheath removed) was unsuccessful as the needle failed to traverse the fibrotic septum.

**Fig. 4:**
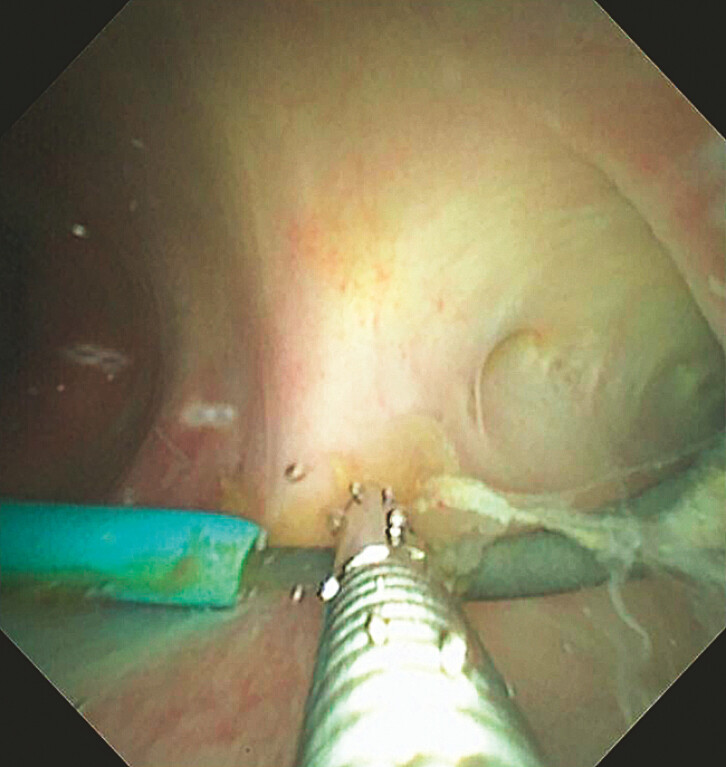
Antegrade cholangioscopic retrieval of the guidewire through the PTCD tract and stent placement. PTCD, percutaneous transhepatic cholangiography and drainage.

**Fig. 5:**
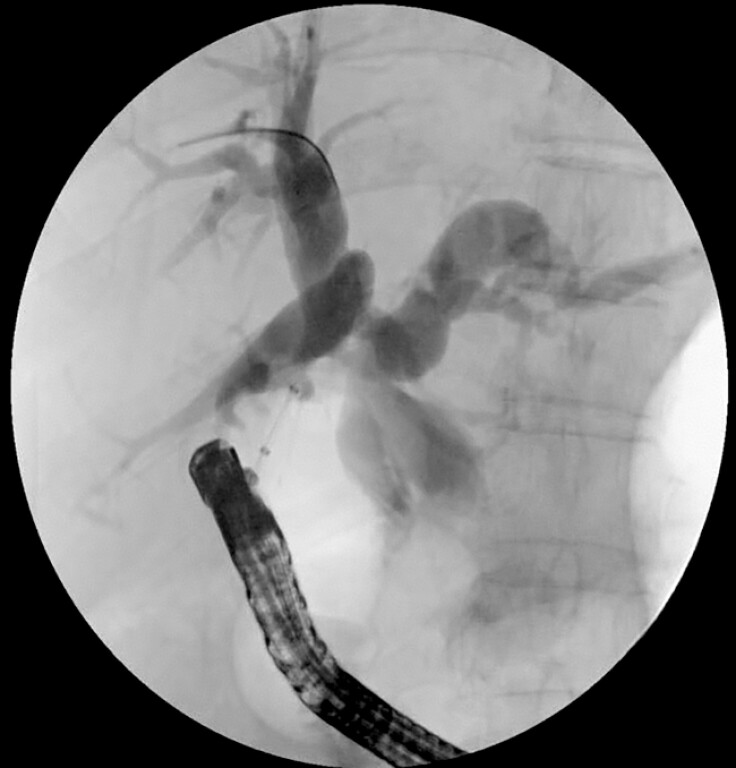
Stent replacement and removal for complete biliary continuity restoration.

A hybrid EUS–PTCD rendezvous approach was then attempted ( Video 1 ). With the modified needle being retained as a radiopaque and sonographically visible marker, the corresponding bile duct segment was precisely localized from the duodenal side, enabling a retrograde EUS puncture to be performed in real time under combined ultrasound and fluoroscopic guidance. Under combined EUS and fluoroscopic guidance, a 19-G EUS needle was advanced from the duodenum toward the right hepatic duct. The guidewire was subsequently advanced retrogradely through the PTCD orifice. A 5-Fr cystotome was introduced along the guidewire to create an incision, followed by the insertion of a nasopancreatic drainage tube to serve as a support channel. Antegrade cholangioscopy through the PTCD tract identified the guidewire, which was grasped and exteriorized with biopsy forceps ( Fig. 4 ). The assistant maintained guidewire tension on the guidewire, and two 7-Fr double-pigtail plastic stents were placed retrogradely to restore internal drainage and anatomical continuity. Biliary drainage was immediately effective, with serum bilirubin levels normalizing within 1 week, and the patient remained asymptomatic. Three-month follow-up cholangiography showed patent stents without bile leakage or infection. The stents were replaced at 6 months and removed after 12 months, achieving complete restoration of biliary continuity ( Fig. 5 ).

Endoscopic ultrasound-guided dual-route recanalization of isolated hepatic ducts following complete hilar disconnection.Video 1

Our group previously reported a hybrid endoscopic approach for recanalizing a completely occluded biliary-enteric anastomosis with impacted stones, demonstrating that EUS-HGS provides a stable platform for complex biliary reconstruction 4 . This case further extends the role of interventional EUS, showing that dual-route recanalization is achievable even in complete hilar disconnection. By combining transluminal and percutaneous routes, this hybrid technique restores biliary continuity, effectively draining the system and avoiding the need for surgical reintervention.

Endoscopy_UCTN_Code_TTT_1AR_2AK
